# Supplementary material for: Four-year nationwide incidence of retinitis pigmentosa in South Korea: a population-based retrospective study from 2011 to 2014
Source: BMJ Open. 2017 May 9;7(5):e015531. doi: 10.1136/bmjopen-2016-015531 (PMC5623391; doi:10.1136/bmjopen-2016-015531)
Supplement: Supplementary data [file bmjopen-2016-015531supp001.pdf]

***Prevalence of Presumed Retinitis Pigmentosa in South Korea***

The Korean Classification of Disease, version 5 was used until 2010, and its classification system rendered it impossible to distinguish Stargardt disease, a disease that shares the same co-payment policy code (V209), from retinitis pigmentosa (RP). Beginning in 2011, the Korean Classification of Disease, version 6 was used, and it enables the discrimination of these two diseases based on the diagnostic code used (H35.51 for RP and H35.58 for Stargardt disease). Therefore, we excluded patients with chronic RP or Stargardt disease from the 2007 (the first year that the co-payment policy started) through 2010 data, and new cases from 1 January 2011 were evaluated to determine the incidence of RP. In our exploratory analysis, there were 27 patients with Stargardt disease diagnosed in 2011, 26 diagnosed in 2012, 29 diagnosed in 2012, and 27 diagnosed in 2014 in South Korea. One hundred nine patients with Stargardt disease was relatively small compared to the 3144 patients with RP diagnosed over the same period. Therefore, to calculate the prevalence, we arbitrarily defined the presumed number of patients with RP between 2007 and 2010 based on the aforementioned incidence ratio of 109/3144. The period prevalence of presumed RP per 100 000 persons was estimated based on data from the Population and Housing Census conducted in 2010.

Overall, 4 428 patients with RP and Stargardt disease were identified between 2007 and 2010, and the presumed number of patients with RP was calculated to be 4 280 [= 4 428 × 3 144 / (3 144 + 109)]. Among 4 428 patients identified as having RP and Stargardt disease, 863 presumed patients with RP were diagnosed in 2007, 1359 in 2008, 1 228 in 2009, and 978 in 2010. As the co-payment policy was established in 2007 and chronic patients as well as new patients started to register from 2007, there was a larger number of patients registered in 2008 and 2009 than in 2010. A total of 3 144 patients with RP were identified between 2011 and 2014. Finally, 7 424 presumed patients with RP (n = 4 280) and RP (n = 3 144) were identified and used to estimate the prevalence. The prevalence of presumed RP was 15.47 cases per 100 000 persons from 2007 to 2014 (table S1). The prevalence was similar between men (15.64 cases/100 000 persons) and women (15.30 cases/100 000 persons). The 50–54-year-old age group had the largest number of patients with RP with 897 prevalent cases. However, the age-specific prevalence was highest in the 65–69-year-old age group with 30.90 cases/100 000 persons.

In the 1990s, several investigators reported an RP prevalence ranging from 12 (1:8247) to 26 cases (1:3784) per 100 000 persons based on well-defined population studies.[1-4] The RP prevalence in the present study, 15.47 cases/100 000 persons, was smaller than that seen in the Danish cohort with RP (22.4 cases/100 000 persons). However, trends in the age-specific prevalence were similar, showing increasing prevalence with age until 70 years old and then a decreasing prevalence.[5] Other recent studies have also reported prevalence; however, some were limited because of the small number of patients with RP.[6-9] In summary, the prevalence of RP, including presumed patients with RP diagnosed between 2007 and 2010, and RP diagnosed between 2011 and 2014 was 15.47 cases/100 000 (1/6500) persons. These data are within the prevalence range reported in previous well-designed studies and slightly lower than that observed in the Danish cohort with RP.

**References**


1. Bunker CH, Berson EL, Bromley WC, Hayes RP, Roderick TH. Prevalence of retinitis pigmentosa in Maine. Am J Ophthalmol. 1984;97(3):357-365.
2. Bundey S, Crews SJ. A study of retinitis pigmentosa in the City of Birmingham. I Prevalence. J Med Genet. 1984;21(6):417-420.
3. Grondahl J. Tapeto-retinal degeneration in four Norwegian counties, II. Diagnostic evaluation of 407 relatives and genetic evaluation of 87 families. Clin Genet. 1986;29(1):17-41.
4. Hu DN. Prevalence and mode of inheritance of major genetic eye diseases in China. J Med Genet. 1987;24(10):584-588.
5. Haim M. Epidemiology of retinitis pigmentosa in Denmark. Acta Ophthalmol Scand Suppl. 2002(233):1-34.
6. You QS, Xu L, Wang YX, et al. Prevalence of retinitis pigmentosa in North China: the Beijing Eye Public Health Care Project. Acta Ophthalmol. 2013;91(6):e499-500.
7. Nangia V, Jonas JB, Khare A, Sinha A. Prevalence of retinitis pigmentosa in India: the Central India Eye and Medical Study. Acta Ophthalmol. 2012;90(8):e649-650.
8. Xu L, Hu L, Ma K, Li J, Jonas JB. Prevalence of retinitis pigmentosa in urban and rural adult Chinese: The Beijing Eye Study. Eur J Ophthalmol. 2006;16(6):865-866.
9. Sen P, Bhargava A, George R, et al. Prevalence of retinitis pigmentosa in South Indian population aged above 40 years. Ophthalmic Epidemiol. 2008;15(4):279-281.


**Table S1** Period Prevalence Per 100 000 Persons of Presumed Retinitis Pigmentosa in the Korean Population from 2007 to 2014

| | Total | | | Men | | | Women | | |
|---|---|---|---|---|---|---|---|---|---|
| Age (years) | No. | Prevalence (95% CI) | | No. | Prevalence (95% CI) | | No. | Prevalence (95% CI) | |
| 0-4 | 25 | 1.13 | 0.73 - 1.66 | 18 | 1.58 | 0.93 - 2.49 | 8 | 0.74 | 0.32 - 1.46 |
| 5-9 | 84 | 3.51 | 2.80 - 4.34 | 50 | 4.02 | 2.98 - 5.30 | 34 | 2.95 | 2.05 - 4.13 |
| 10-14 | 118 | 3.72 | 3.08 - 4.45 | 76 | 4.59 | 3.62 - 5.75 | 42 | 2.77 | 1.99 - 3.74 |
| 15-19 | 320 | 9.31 | 8.31 - 10.38 | 231 | 12.65 | 11.07 - 14.39 | 89 | 5.52 | 4.43 - 6.79 |
| 20-24 | 347 | 11.36 | 10.19 - 12.62 | 229 | 14.09 | 12.32 - 16.04 | 119 | 8.32 | 6.89 - 9.96 |
| 25-29 | 384 | 10.85 | 9.79 - 11.99 | 210 | 11.65 | 10.13 - 13.34 | 174 | 10.02 | 8.59 - 11.63 |
| 30-34 | 479 | 12.96 | 11.83 - 14.18 | 270 | 14.47 | 12.79 - 16.30 | 209 | 11.43 | 9.93 - 13.09 |
| 35-39 | 558 | 13.61 | 12.51 - 14.79 | 297 | 14.42 | 12.82 - 16.15 | 261 | 12.80 | 11.29 - 14.45 |
| 40-44 | 658 | 15.93 | 14.73 - 17.19 | 348 | 16.80 | 15.08 - 18.66 | 310 | 15.05 | 13.42 - 16.82 |
| 45-49 | 747 | 18.34 | 17.05 - 19.70 | 392 | 19.17 | 17.32 - 21.17 | 355 | 17.50 | 15.73 - 19.42 |
| 50-54 | 897 | 23.62 | 22.10 - 25.21 | 441 | 23.36 | 21.23 - 25.64 | 456 | 23.87 | 21.73 - 26.17 |
| 55-59 | 721 | 26.06 | 24.19 - 28.03 | 339 | 24.91 | 22.33 - 27.71 | 382 | 27.17 | 24.51 - 30.04 |
| 60-64 | 651 | 29.83 | 27.58 - 32.21 | 286 | 27.06 | 24.01 - 30.38 | 366 | 32.53 | 29.28 - 36.04 |
| 65-69 | 560 | 30.90 | 28.40 - 33.57 | 220 | 26.40 | 23.03 - 30.13 | 339 | 34.63 | 31.04 - 38.52 |
| 70-74 | 436 | 27.84 | 25.29 - 30.58 | 174 | 25.86 | 22.16 - 30.00 | 262 | 29.34 | 25.89 - 33.11 |
| 75-79 | 266 | 24.53 | 21.67 - 27.66 | 94 | 22.89 | 18.49 - 28.01 | 171 | 25.38 | 21.72 - 29.49 |
| 80-84 | 131 | 22.00 | 18.39 - 26.10 | 42 | 22.58 | 16.27 - 30.52 | 89 | 21.73 | 17.45 - 26.75 |
| ≥85 | 42 | 11.46 | 8.26 - 15.49 | 14 | 14.78 | 8.08 - 24.79 | 28 | 10.30 | 6.84 - 14.88 |
| Total | 7 424 | 15.47 | 15.12 - 15.83 | 3 729 | 15.64 | 15.14 - 16.15 | 3 695 | 15.30 | 14.81 - 15.80 |

CI = confidence interval, no. = number
