# Supplementary material for: Four-year nationwide incidence of retinitis pigmentosa in South Korea: a population-based retrospective study from 2011 to 2014
Source: BMJ Open. 2017 May 9;7(5):e015531. doi: 10.1136/bmjopen-2016-015531 (PMC5623391; doi:10.1136/bmjopen-2016-015531)
Supplement: Author's manuscript [file bmjopen-2016-015531.draft_revisions.pdf]

BMJ Open

Four-Year Nationwide Incidence of Retinitis Pigmentosa in South Korea: A Population-based Retrospective Study from 2011 to 2014

Journal:	*BMJ Open*
Manuscript ID	bmjopen-2016-015531
Article Type:	Research
Date Submitted by the Author:	13-Dec-2016

Complete List of Authors:	Rim, Tyler Hyung Taek; Severance Hospital, Institute of Vision Research, Yonsei University College of Medicine, Department of Ophthalmology<br>Park, Hye Won ; Severance Hospital, Institute of Vision Research, Yonsei University College of Medicine, Department of Ophthalmology<br>Kim, Dong Wook ; National Health Insurance Service Ilsan Hospital<br>Chung, Eun Jee ; National Health Insurance Service Ilsan Hospital

**Primary Subject Heading**:	Epidemiology
Secondary Subject Heading:	Ophthalmology, Emergency medicine
Keywords:	retinitis pigmentosa, south korea, Asian population

SCHOLARONE™ Manuscripts

For peer review only

Four-Year Nationwide Incidence of Retinitis Pigmentosa in South Korea: A Population-based Retrospective Study from 2011 to 2014

Tyler Hyungtaek Rim,[1*] M.D., M.B.A, Hye Won Park,[1,2*] M.D., Dong Wook Kim,[3] Ph.D., Eun Jee Chung,[1] M.D., Ph.D.

[1]Department of Ophthalmology, National Health Insurance Service Ilsan Hospital, Goyang, Korea [2]Department of Ophthalmology, Severance Hospital, Institute of Vision Research, Yonsei University College of Medicine, Seoul, Korea; [3]Department of Policy Research Affairs, National Health Insurance Service Ilsan Hospital, Goyang, Gyeonggi-do, Korea;

[*]Contributed equally

Corresponding Author: Eun Jee Chung M.D., Ph.D.

Department of Ophthalmology, National Health Insurance Service Ilsan Hospital

#100 Ilsan-ro, Ilsandong-gu, Goyang 10444, Gyeonggi-do, Korea

Tel: 82-31-900-0590, Fax: 82-31-900-0049

E-mail: eunjee95@nhimc.or.kr

Text Word Count: 2525

Funding Support: This work was supported by a National Health Insurance Ilsan Hospital grant (NHIMC 2015-02-015).

For peer review only

Abstract

Objective: To determine the incidence of retinitis pigmentosa (RP) in South Korea.

Design: Nationwide population-based retrospective study.

Setting: Census population of South Korea

Participants: This study involved the entire population of South Korea (n = 47 990 761). Patients confirmed as having RP by an ophthalmologist from January 1, 2011 to December 31, 2014 were included.

Primary outcome measure: The average incidence of RP during the 4-year study period was estimated using population data from the 2010 Korean census.

Results: A total of 3 144 (1 567 men and 1 577 women) confirmed RP patients were identified. The average incidence of RP was 1.64 cases/100 000 person-years (95% confidence interval, 1.58–1.70). The incidence of RP distribution skewed to the left across age groups, with one smaller peak observed in the 20–24-year-old age group (1.24 cases/100 000 person-years) and a larger peak observed in the 65–69-year-old age group (3.26 cases/100 000 person-years). The overall incidence was similar in men and women (1.64 cases/100 000 person-years [95% confidence interval, 1.56–1.73] for men; 1.63 cases/100 000 person-years [95% confidence interval, 1.55–1.72] for women).

Conclusions: Our study's estimates of the nationwide population-based incidence of RP in an Asian population will help advance the understanding of the disease onset and allow healthcare systems to plan accordingly.

For peer review only - http://bmjopen.bmj.com/site/about/guidelines.xhtml

Strengths and limitations of this study

- In this retrospective cohort study using RP registry data for the entire South Korean population, the incidence of RP was found to be 1.64 cases per 100,000 person-years.
- This study is unique because it included the largest population ever studied in terms of this rare disease as well as the fact that it represents the first investigation of the nationwide incidence of RP in an Asian country.
- Only a few recent studies have collected data for a general population and calculated the incidence of retinitis pigmentosa (RP). There are no nationwide RP-related epidemiological studies.
- We might have underestimated the incidence rates of RP and a lack of clinical information including family pedigree, visual acuity, visual field, and genetic analysis, is another inherent limitation of the present claims database study.
- While not ideal, our approach was cost-effective for identifying RP cases and calculating the incidence of RP in the entire South Korean population.

For peer review only

For peer review only - http://bmjopen.bmj.com/site/about/guidelines.xhtml

Introduction

Retinitis pigmentosa (RP) includes a group of heterogeneous hereditary retinal degeneration disorders caused by the progressive loss of photoreceptor cells.[1 2] It is characterised by widespread retinal degeneration and is an important and leading cause of blindness.[3] However, there is a paucity of data based on large, well-defined populations regarding the epidemiological characteristics of RP. Ideally, a study evaluating RP epidemiology should be based on population data rather than data obtained from a tertiary referral centre to avoid the effects of a referral bias. To the best of our knowledge, only a few recent studies have collected data from a general population and calculated the incidence of RP. RP incidence was reported in only two previous studies conducted in Denmark (0.79 cases/100 000 person-years)[4] and Maine in the United State (6 cases/100 000 person-years).[5] Despite recent large-scale health-related studies[6 7]; there are no nationwide RP-related epidemiological studies. In general, incidence estimates provide more information than prevalence estimates with regard to a disease's characteristics and for helping healthcare systems plan accordingly.

South Korea has a mandatory universal health insurance system covering the entire population of 48 million people; therefore, the medical claims database includes all healthcare use in South Korea. In addition, the Korean government initiated a registration program for 'rare and intractable disorders', such as RP, in 2007. RP patients registered in this program are eligible for up to a 90% co-payment reduction after the diagnosis has been confirmed by an ophthalmologist using the Korean National Health Insurance Service (KNHIS) diagnostic criteria based on electrophysiological examination. Our aim was to use this database to conduct a nationwide population-based study to determine the incidence of RP in South Korea.

For peer review only - http://bmjopen.bmj.com/site/about/guidelines.xhtml

Subjects and Methods

Statement of Ethics

This retrospective nationwide cohort study was approved by our institutional review board, which waived the requirement for informed consent.

Retinitis pigmentosa registration in South Korea and retinitis pigmentosa definition

All Korean residents are obligated to enrol in the KNHIS. Claims are accompanied by data regarding diagnostic codes, procedures, prescription drugs, personal information, and information about the hospital. No patient health care records are duplicated because all Korean residents receive a unique identification number at birth. Furthermore, the KNHIS uses the *Korean Classification of Diseases*, which is a system similar to the *International Classification of Diseases*. In 2007, the National Health Insurance Service initiated a co-payment reduction of up to 90% for patients suffering from 138 rare and intractable disorders, including RP. Patients with RP who registered in the program were eligible for co-payment reduction after a confirmed diagnosis by an ophthalmologist based on the National Health Insurance Service diagnostic criteria. The National Health Insurance Service diagnostic criteria for RP require (1) ophthalmoscopic abnormalities of the retina on a dilated fundus examination and (2) electroretinographic changes confirming the presence of RP-related photoreceptor damage. All submissions for co-payment reduction registration were reviewed and confirmed by the Health Insurance Review Agency. Patients must reapply for co-payment reduction registration every 5 years after the initial registration to maintain the co-payment reduction. After registration, all RP related claims contain the RP registration code (V209) in addition to the diagnostic code for RP (Korean Classification of Diseases H3551). Patients who filed claims for RP (V209, H3551) from January 2011 to December 2014 with the KNHIS were included in this study. We excluded chronic RP patients registered between

For peer review only - http://bmjopen.bmj.com/site/about/guidelines.xhtml

January 1, 2007 and December 31, 2010, because RP (H3551) and Stargardt's disease (H3558) shared the same registration code (V209) without a Korean Classification of Diseases diagnostic code in the KNHIS database during this time. Finally, we obtained RP registration data from the national health claim database between 2007 and 2014 and calculated the incidence of RP between 2011 and 2014.

Statistical analysis

Incident time was defined as the registration date of a 'rare and intractable disorder' diagnosed as RP. Annual population data were obtained from the Population and Housing Census conducted in 2010 and were available from the Korean Statistical Information Service (http://kosis.kr). Detailed demographics of the South Korean population are listed in table 1. The person-time incidence rates for 2011–2014 were calculated as the number of people who developed RP divided by the total person-time at risk during the study period. Therefore, in this analysis, person-years were counted after the incident time. Incidence per 100 000 person-years, based on the 2010 census, was estimated using the Poisson distribution. In our explorative analysis (supplementary digital content 1, appendix) the total number of RP cases from 2007 to 2014 was estimated at 7 424. Prevalent RP cases were not excluded from the population at risk because the denominator (total Korean person-time at risk) was large enough to not be affected in terms of incidence. Total person-time at risk was assumed as the total population (Census in 2010, N=47 990 761, table 1) over 4 years from 2011 to 2014 (population multiplied by four). Annual incidence from 2011 to 2014 and overall incidence were estimated with 95% confidence intervals and the age- and sex-specific incidence rates were estimated. The male-to-female ratio for the RP incidence rate was also estimated. A significance level of 0.05 was selected. All analyses were conducted using Stata/MP, version 14.0 (StataCorp, College Station, TX, USA).

For peer review only

For peer review only - http://bmjopen.bmj.com/site/about/guidelines.xhtml

Results

The total number of incident RP cases from 2011 to 2014 in South Korea was 3,144, including 1 567 men (49.9%) and 1 577 women (50.1%). The incidence of RP during the study period was 1.64 cases/100 000 person-years. In men, the incidence was 1.64 cases/100 000 person-years and in women, it was 1.63 cases/100 000 person-years. The peak incidence in the total population was observed in the 65–69-year-old age group (3.26 cases/100 000 person-years; men, 3.00 cases/100 000 person-years; women, 3.86 cases/100 000 person-years) (table 1 and figure 1). Although there was some variation between the different time periods and age groups, the overall incidences in men and women were similar (male to female ratio = 1.01, p = 0.80). The incidence of RP distribution was negatively skewed (skewed to the left) across age groups, with one smaller peak observed in 20–24 year olds (1.24 cases/100 000 person-years). The incidence was slightly decreased in the 25–29-year-old age group (1.07 cases/100 000 person-years) and the maximum incidence was seen in the 65–69-year-old age group (3.26 cases/100 000 person-years) (figure 1). The incidence for women steadily increased with age, reaching a maximum level in the 65–69-year-old age group, and then gradually decreased after age 69. There was a unique peak in incidence for men around 20 years of age (figure 1).

There were 851 (27.1%), 708 (22.5%), 728 (23.2%), and 857 (27.3%) incident cases in 2011, 2012, 2013, and 2014, respectively. There was little difference in the annual number of newly diagnosed RP cases from 2011 to 2014. The annual incidence of RP was slightly higher in 2011 and 2014 and slightly lower in 2012 and 2013 (1.77 cases/100 000 population in 2011, 1.48 cases/100 000 population in 2012, 1.52 cases/100 000 population in 2013, and 1.79 cases/100 000 population in 2014, table 2).

For peer review only - http://bmjopen.bmj.com/site/about/guidelines.xhtml

Discussion

In the present study, using the KNHIS database between 2011 and 2014, we found a nationwide, population-based, estimated RP incidence of 1.64 cases/100 000 person-years. This study involved a survey of the entire South Korean population using reliable data based on an ophthalmologist-confirmed RP registration.

RP is not an acute symptomatic disease like retinal vascular occlusion. The most common initial symptom of RP, night blindness, has an insidious onset and may go unperceived for many years. Often, patients become cognizant of RP incidentally. Therefore, previous studies from Japan noted the disease onset as the time of a diagnosis of RP by an ophthalmologist.[8] Another study from Denmark discussed possible recall bias, and cases were classified as possible, probable, or certain according to the presence of specified diagnostic criteria and confirmatory electroretinographic changes. RP is very rare; therefore, it is impossible to estimate the incidence without a large cohort of subjects, such as that provided by nationwide data. However, it is practically impossible to carry out ophthalmological examinations of the entire population to diagnosis a rare intractable disease such as RP. Our study design is based on the ophthalmologist-confirmed RP registry and demonstrates a high specificity for the diagnosis. While not ideal, our approach was cost-effective for identifying RP cases and calculating the incidence of RP in the entire South Korean population. Studying a large cohort, such as the entire South Korean population, and having a 4-year study period may provide stability to the heterogeneous detection rate seen with this insidious disease. These properties limit the chance probabilities that may occur with studies of smaller localised populations or tertiary hospital based populations. Studies regarding the incidence of other rare intractable diseases, as exudative age-related macular degeneration[6] and Moyamoya disease,[7] using the same registration database, have been previously reported.

For peer review only - http://bmjopen.bmj.com/site/about/guidelines.xhtml

Using the South Korean national claims database from 2011 to 2014, which includes the RP registration program, we estimated the incidence rate of RP as 1.64 cases/100 000 person-years [95% confidence interval, 1.58–1.70]. To the best of our knowledge, only two previous studies have estimated the incidence of RP based on an adequate number of RP patients. A well-defined local area study conducted in the 1980s in the state of Maine (USA) estimated that the incidence of RP was 6 cases/100 000 person-years.[5] Another important previous study regarding RP epidemiology, performed in Denmark in the 2000s, reported an average incidence of 0.79 cases/100 000 person-years from 1990 to 1997.[4] The incidence of RP in South Korea found in the present study is less than that seen in Maine and greater than that seen in Denmark.

Information regarding the age of disease onset is meaningful in genetic counselling, and a previous study from Japan described an age at onset curve for RP based on 370 RP patients.[8] The age at onset, defined as the age of diagnosis, gradually increased with age until age 65 years and subsequently remained steady after age 65.[8] In the Danish RP study,[4] the incidence of RP also increased until age 60–64 years, and then decreased in people older than 65 years old, similar to the trend seen in the present study (figure 1). In terms of sex, the Danish RP cohorts showed a nearly equal increase in the annual incidence between men and women from 1990 to 1997.[4] The present study showed similar trends; the overall male to female ratio was 1.01 (p = 0.86) and the annual incidence of RP was similar for men and women during the 4-year study period (table 2). However, the estimated age-specific incidence of newly diagnosed cases was nearly double in male patients compared with that of female patients in the 20–24-year-old age group (men, 1.54 cases/100 000 population and women, 0.89 cases/100 000 population) (table 1). In addition, the incidence of RP in women was greater than that of men in 50–65 year olds. In the Danish RP study, the age at onset was much

For peer review only - http://bmjopen.bmj.com/site/about/guidelines.xhtml

higher in males than that seen in females aged 6–18 years old.[4] Our results also show that the onset may be earlier in males compared to that seen in females. However, the specifics of military service in South Korea also should be considered. The higher incidence of RP in men in their 20s, entering mandatory military service in South Korea, may be because they are more frequently subjected to physical examinations related to potential exemption from military service.

To the best of our knowledge, this is the largest population ever studied for this rare disease based on an ophthalmologist confirmed diagnosis. It is also the first study to investigate the nationwide incidence of RP in an Asian country.

However, there are several limitations of this study. First, as we mentioned above, it is difficult to define the exact time of onset of RP, and we arbitrary defined the time of occurrence of RP as the time when the patients were diagnosed with RP by an ophthalmologist, as in previous studies.[4 8] Second, we might have underestimated the incidence rates of RP. We identified RP cases using healthcare claims and could not include asymptomatic patients or patients who did not use healthcare services. Since there is no cure for RP as yet, some patients might not want to register, even though they were diagnosed with RP. However, it is reasonable to assume that people with symptomatic RP are likely to use healthcare services at some time in the course of the disease. The universal health insurance coverage and co-payment reduction with the registration program for this rare and intractable disorder may also help to encourage healthcare use in patients with RP. Moreover, healthcare accessibility in South Korea is very high. The estimated incidence of newly diagnosed cases showed a consistent annual pattern and the relatively long study period, including 4 years of data, might offset this limitation. Third, the diagnosis of RP was defined on the basis of healthcare registration and a *Korean Classification of Diseases* code, which may be

For peer review only - http://bmjopen.bmj.com/site/about/guidelines.xhtml

inaccurate compared with a diagnosis obtained from a medical record. However, RP patients in this study had their RP diagnoses confirmed by ophthalmologists based on the National Health Insurance Service diagnostic criteria including electroretinography, a test that is considered as a confirmatory criteria.[4] In addition, the eligibility of submitted registrations was reviewed comprehensively by the Health Insurance Review Agency. Fourth, a lack of clinical information including family pedigree, visual acuity, visual field, and genetic analysis, is another inherent limitation of the present claims database study. Fifth, we only targeted subjects in South Korea. Therefore, this result cannot be directly compared with those of other ethnic groups. Lastly, we did not report the prevalence of RP because of the characteristics of the South Korean national claims database. The analysis of prevalence for RP was impossible with the Korean Classification of Disease, sixth edition (*Korean Classification of Diseases*-6) system, in which RP, Stargardt's disease, vitelliform retinal dystrophy, and other unspecified hereditary retinal dystrophies were similarly categorised as hereditary retinal dystrophy (H35.5). In this study, we excluded RP subjects registered prior to December 31, 2010, because it was difficult to distinguish among these diseases in the KNHIS database. In our explorative analysis, only 109 incident cases of Stargardt's disease were observed from 2011 to 2014, a relatively small number compared to the 3,144 cases of RP. Therefore, we have presented the period prevalence of presumed RP via a supplementary digital content 1, appendix showing a prevalence of 15.47 cases/100 000 persons, a value consistent with previous reports (12–26 cases/100 000 persons)[5 9-11] and slightly less than that found in Danish RP cohorts (22.4 cases/100 000 persons).[4]

In conclusion, we estimated the nationwide incidence of newly diagnosed RP cases in South Korea using a database that included the entire national population during the 4-year period from 2011 to 2014. The estimated population-based incidence rate for RP for all ages

For peer review only - http://bmjopen.bmj.com/site/about/guidelines.xhtml

was 1.63 cases/100 000 persons, and there was generally no difference in the rates between men and women. Our study should be helpful in assessing the RP-related socioeconomic burden and in planning accordingly within the healthcare system.

For peer review only

For peer review only - http://bmjopen.bmj.com/site/about/guidelines.xhtml

Acknowledgments

This work was supported by a National Health Insurance Ilsan Hospital grant (NHIMC 2015-02-015). This study used data from the NHIS-NCS 2002-2013 (NHIS-2015-1-068), which was released by the KNHIS. The authors alone are responsible for the content and writing of this article. Eun Jee Chung had full access to all of the data in the study and takes responsibility for the integrity of the data and the accuracy of the data analysis.

Competing Interests: We have read and understood BMJ policy on declaration of interests and declare that we have no competing interests.

Authors' Contributions:

Conception and design: THR and EJC

Analysis and interpretation: THR, HWP, DWK, and EJC

Data collection: THR, HWP, DWK, and EJC

Manuscript preparation: THR and HWP

Overall responsibility: EJC

For peer review only

References

1. Carr RE, Noble KG. Retinitis pigmentosa. *Ophthalmology* 1981;88(2):169-72.
2. Hartong DT, Berson EL, Dryja TP. Retinitis pigmentosa. *Lancet* 2006;368(9549):1795-809. doi: 10.1016/S0140-6736(06)69740-7
3. Buch H, Vinding T, La Cour M, et al. Prevalence and causes of visual impairment and blindness among 9980 Scandinavian adults: the Copenhagen City Eye Study. *Ophthalmology* 2004;111(1):53-61.
4. Haim M. Epidemiology of retinitis pigmentosa in Denmark. *Acta Ophthalmol Scand Suppl* 2002(233):1-34.
5. Bunker CH, Berson EL, Bromley WC, et al. Prevalence of retinitis pigmentosa in Maine. *Am J Ophthalmol* 1984;97(3):357-65.
6. Park SJ, Kwon KE, Choi NK, et al. Prevalence and Incidence of Exudative Age-Related Macular Degeneration in South Korea: A Nationwide Population-Based Study. *Ophthalmology* 2015;122(10):2063-70 e1. doi: 10.1016/j.ophtha.2015.06.018
7. Ahn IM, Park DH, Hann HJ, et al. Incidence, prevalence, and survival of moyamoya disease in Korea: a nationwide, population-based study. *Stroke* 2014;45(4):1090-5. doi: 10.1161/STROKEAHA.113.004273
8. Tsujikawa M, Wada Y, Sukegawa M, et al. Age at onset curves of retinitis pigmentosa. *Archives of ophthalmology* 2008;126(3):337-40.
9. Bundey S, Crews SJ. A study of retinitis pigmentosa in the City of Birmingham. I Prevalence. *J Med Genet* 1984;21(6):417-20.
10. Grondahl J. Tapeto-retinal degeneration in four Norwegian counties, II. Diagnostic evaluation of 407 relatives and genetic evaluation of 87 families. *Clin Genet* 1986;29(1):17-41.
11. Hu DN. Prevalence and mode of inheritance of major genetic eye diseases in China. *J Med Genet* 1987;24(10):584-8.

For peer review only

For peer review only - http://bmjopen.bmj.com/site/about/guidelines.xhtml

Figure Legend

Figure 1. Average incidence of retinitis pigmentosa (RP). Incidence per 100 000 person-years of RP in the South Korean population according to age groups from 2011 to 2014.

Supplementary Information

Supplementary Digital Content 1, Appendix (Text Document)

For peer review only

For peer review only - http://bmjopen.bmj.com/site/about/guidelines.xhtml

Table 1 Demographics of South Korea and the incidence of retinitis pigmentosa per 100 000 person-years in the South Korean population (2011-2014)

	Number of Korean Population			Total			Men			Women			Male to	P value
Age (years)	Total	Men	Women	No.	Incidence (95% CI)		No.	Incidence (95% CI)		No.	Incidence (95% CI)		Female Ratio	(chi test)
0-4	2 219 084	1 142 220	1 076 864	4	0.05	0.01- 0.12	4	0.09	0.02- 0.22	0	no observation		NA	0.05
5-9	2 394 663	1 243 294	1 151 369	21	0.22	0.14- 0.34	14	0.28	0.15- 0.47	7	0.15	0.06- 0.31	1.86	0.18
10-14	3 173 226	1 654 964	1 518 262	28	0.22	0.15- 0.32	16	0.24	0.14- 0.39	12	0.20	0.10- 0.35	1.22	0.60
15-19	3 438 414	1 826 179	1 612 235	123	0.89	0.74- 1.07	92	1.26	1.02- 1.54	31	0.48	0.33- 0.68	2.62	<0.05
20-24	3 055 420	1 625 371	1 430 049	151	1.24	1.05- 1.45	100	1.54	1.25- 1.87	51	0.89	0.66- 1.17	1.73	<0.05
25-29	3 538 949	1 802 805	1 736 144	152	1.07	0.91- 1.26	84	1.16	0.93- 1.44	68	0.98	0.76- 1.24	1.18	0.29
30-34	3 695 348	1 866 397	1 828 951	209	1.41	1.23- 1.62	112	1.50	1.24- 1.81	97	1.33	1.08- 1.62	1.13	0.37
35-39	4 099 147	2 060 233	2 038 914	239	1.46	1.28- 1.65	129	1.57	1.31- 1.86	110	1.34	1.10- 1.61	1.17	0.25
40-44	4 131 423	2 071 431	2 059 992	307	1.86	1.66- 2.08	162	1.96	1.67- 2.28	145	1.76	1.48- 2.07	1.11	0.36
45-49	4 073 358	2 044 641	2 028 717	304	1.87	1.66- 2.09	148	1.81	1.53- 2.13	156	1.92	1.63- 2.25	0.94	0.60
50-54	3 798 131	1 887 973	1 910 158	405	2.67	2.41- 2.94	204	2.70	2.34- 3.10	201	2.63	2.28- 3.02	1.03	0.79
55-59	2 766 695	1 360 747	1 405 948	327	2.95	2.64- 3.29	150	2.76	2.33- 3.23	177	3.15	2.70- 3.65	0.88	0.23
60-64	2 182 236	1 057 035	1 125 201	283	3.24	2.88- 3.64	127	3.00	2.50- 3.57	156	3.47	2.94- 4.05	0.86	0.23
65-69	1 812 168	833 242	978 926	236	3.26	2.85- 3.70	85	2.55	2.04- 3.15	151	3.86	3.27- 4.52	0.66	<0.05
70-74	1 566 014	672 894	893 120	178	2.84	2.44- 3.29	72	2.68	2.09- 3.37	106	2.97	2.43- 3.59	0.90	0.50
75-79	1 084 367	410 726	673 641	108	2.49	2.04- 3.01	49	2.98	2.21- 3.94	59	2.19	1.67- 2.82	1.36	0.11
80-84	595 509	186 008	409 501	56	2.35	1.78- 3.05	13	1.75	0.93- 2.99	43	2.63	1.90- 3.54	0.67	0.20
≥85	366 609	94 736	271 873	13	0.89	0.47- 1.52	6	1.58	0.58- 3.45	7	0.64	0.26- 1.33	2.45	0.09
Total	47 990 761	23 840 896	24 149 865	3144	1.64	1.58- 1.70	1567	1.64	1.56- 1.73	1577	1.63	1.55- 1.72	1.01	0.86

The population of Korea was based on the 2010 census from the Korean Statistical Information Service. NA = not available, CI = confidence interval.

For peer review only - http://bmjopen.bmj.com/site/about/guidelines.xhtml

Table 2 Demographics of South Korea and the annual incidence of retinitis pigmentosa per 100 000 person-years in the South Korean population (2011-2014)

	2011			2012			2013			2014		
Age (years)	Total	Men	Women	Total	Men	Women	Total	Men	Women	Total	Men	Women
0-4	0.05	0.09	0.00	0.05	0.09	0.00	0.05	0.09	0.00	0.05	0.09	0.00
5-9	0.21	0.40	0.00	0.33	0.40	0.26	0.17	0.08	0.26	0.17	0.24	0.09
10-14	0.13	0.24	0.00	0.25	0.18	0.33	0.25	0.36	0.13	0.25	0.18	0.33
15-19	1.16	1.75	0.50	0.84	1.15	0.50	0.73	0.88	0.56	0.84	1.26	0.37
20-24	1.44	1.66	1.19	0.88	1.11	0.63	1.15	1.48	0.77	1.47	1.91	0.98
25-29	1.47	1.61	1.32	0.88	0.94	0.81	0.93	1.22	0.63	1.02	0.89	1.15
30-34	1.41	1.23	1.59	1.19	1.34	1.04	1.49	1.71	1.26	1.57	1.71	1.42
35-39	1.68	1.84	1.52	1.22	1.26	1.18	1.59	1.70	1.47	1.34	1.46	1.23
40-44	1.65	1.54	1.75	1.94	2.17	1.70	1.69	1.79	1.60	2.15	2.32	1.99
45-49	1.77	1.71	1.82	1.69	1.91	1.48	1.72	1.37	2.07	2.28	2.25	2.32
50-54	3.37	3.07	3.66	2.47	2.65	2.30	2.24	2.28	2.20	2.58	2.81	2.36
55-59	3.04	3.01	3.06	2.42	2.35	2.49	2.82	2.79	2.85	3.54	2.87	4.20
60-64	3.39	2.93	3.82	3.30	3.41	3.20	3.07	2.74	3.38	3.21	2.93	3.47
65-69	3.92	2.76	4.90	2.70	2.28	3.06	2.98	1.92	3.88	3.42	3.24	3.58
70-74	3.00	2.97	3.02	2.68	3.27	2.24	2.81	2.08	3.36	2.87	2.38	3.25
75-79	2.21	2.92	1.78	1.75	2.19	1.48	1.94	2.43	1.63	4.06	4.38	3.86
80-84	2.02	1.08	2.44	2.35	0.54	3.17	2.02	3.23	1.47	3.02	2.15	3.42
≥85	1.09	2.11	0.74	1.09	2.11	0.74	0.27	0.00	0.37	1.09	2.11	0.74
Total	1.77	1.74	1.81	1.48	1.56	1.40	1.52	1.50	1.53	1.79	1.77	1.80

For peer review only - http://bmjopen.bmj.com/site/about/guidelines.xhtml

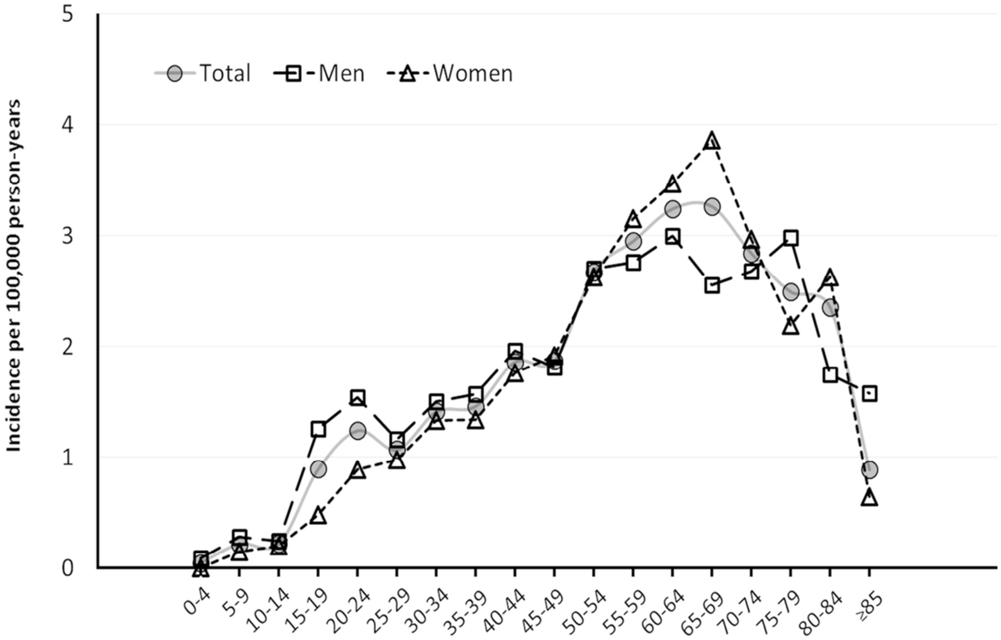

Figure 1. Average incidence of retinitis pigmentosa (RP). Incidence per 100 000 person-years of RP in the South Korean population according to age groups from 2011 to 2014.

51x33mm (600 x 600 DPI)

view only

Prevalence of Presumed Retinitis Pigmentosa in South Korea

The *Korean Classification of Disease* version 5 was used until 2010, and its classification system rendered it impossible to distinguish Stargardt's disease, a disease that shares the same 'co-payment policy' code (V209), from retinitis pigmentosa (RP). Beginning in 2011, *Korean Classification of Disease* version 6 was used and it allows the discrimination of these two diseases based on the diagnostic code used (H35.51 for RP and H35.58 for Stargardt's disease). Therefore, we excluded chronic RP or Stargardt's disease patients from the 2007 (the first year that the copayment policy started) through 2010 data, and new cases from January 1, 2011 were evaluated to determine the incidence of RP. In our exploratory analysis, there were 27 cases of Stargardt's disease diagnosed in 2011, 26 cases diagnosed in 2012, 29 cases diagnosed in 2012, and 27 cases diagnosed in 2014 in South Korea. The total of 109 cases of Stargardt's disease was relatively small compared to the 3144 cases of RP diagnosed over the same time period. Therefore, in order to calculate the prevalence, we arbitrarily defined the presumed number of RP cases between 2007 and 2010 based on the above incidence ratio of 109/3144. The period prevalence of presumed RP per 100 000 persons was estimated based on data from the Population and Housing Census conducted in 2010.

A total of 4 428 RP and Stargardt's disease cases were identified between 2007 and 2010, and the presumed number of RP cases was calculated to be 4 280 [= 4 428×3 144/(3 144 + 109)]. Overall, 863 presumed RP cases were diagnosed in 2007, 1359 cases in 2008, 1 228 cases in 2009, and 978 cases in 2010 among the total 4 428 RP and Stargardt's disease cases identified. Because the 'co-payment policy' was established in 2007, and chronic patients as well as new patients started to register from 2007, there was a larger number of cases registered in 2008 and 2009 than in 2010. A total of 3 144 RP cases were identified between 2011 and 2014. Finally, a total of 7 424 presumed RP (n = 4 280) and RP (n = 3 144) cases were identified and used for estimating the prevalence. The prevalence of presumed RP was 15.47 cases per 100 000 persons from 2007 to 2014 (See table S1). The prevalence was similar between men (15.64 cases/100 000 persons) and women (15.30 cases/100 000 persons). The 50–54-year-old age group had the largest number of RP patients with 897 prevalent cases. However, the age-specific prevalence was highest in the 65-69 year old age group with 30.90 cases/100 000 persons.

In the 1990s, several investigators reported an RP prevalence ranging from 12 (1:8247) to 26 cases (1:3784) per 100 000 persons based on well-defined population studies.[1-4] The RP prevalence in the present study, 15.47 cases/100 000 persons was smaller than that seen in the Danish RP cohort (22.4 cases/100 000 persons). However, trends in age-specific prevalence were similar, showing increasing prevalence with age until age 70, and then decreasing.[5] Other recent studies also reported prevalence; however, some studies were limited because of the small number of RP patients.[6-9]

In summary, the prevalence of RP, including presumed RP cases diagnosed between 2007 and 2010, and RP diagnosed between 2011 and 2014 was 15.47 cases/100 000 persons. These data are within the prevalence range reported in previous well-designed studies and slightly less than that observed in the Danish RP cohort.

References

1. Bunker CH, Berson EL, Bromley WC, Hayes RP, Roderick TH. Prevalence of retinitis pigmentosa in Maine. Am J Ophthalmol. 1984;97(3):357-365.
2. Bundey S, Crews SJ. A study of retinitis pigmentosa in the City of Birmingham. I Prevalence. J Med Genet. 1984;21(6):417-420.
3. Grondahl J. Tapeto-retinal degeneration in four Norwegian counties, II. Diagnostic evaluation of 407 relatives and genetic evaluation of 87 families. Clin Genet. 1986;29(1):17-41.
4. Hu DN. Prevalence and mode of inheritance of major genetic eye diseases in China. J Med Genet. 1987;24(10):584-588.
5. Haim M. Epidemiology of retinitis pigmentosa in Denmark. Acta Ophthalmol Scand Suppl. 2002(233):1-34.
6. You QS, Xu L, Wang YX, et al. Prevalence of retinitis pigmentosa in North China: the Beijing Eye Public Health Care Project. Acta Ophthalmol. 2013;91(6):e499-500.
7. Nangia V, Jonas JB, Khare A, Sinha A. Prevalence of retinitis pigmentosa in India: the Central India Eye and Medical Study. Acta Ophthalmol. 2012;90(8):e649-650.
8. Xu L, Hu L, Ma K, Li J, Jonas JB. Prevalence of retinitis pigmentosa in urban and rural adult Chinese: The Beijing Eye Study. Eur J Ophthalmol. 2006;16(6):865-866.
9. Sen P, Bhargava A, George R, et al. Prevalence of retinitis pigmentosa in South Indian population aged above 40 years. Ophthalmic Epidemiol. 2008;15(4):279-281.

Table S1 Period Prevalence per 100,000 Persons of Presumed Retinitis Pigmentosa in Korean Population from 2007 to 2014

	Total			Men			Women		
Age (years)	No.	Incidence (95% CI)		No.	Incidence (95% CI)		No.	Incidence (95% CI)	
0-4	25	1.13	0.73 - 1.66	18	1.58	0.93 - 2.49	8	0.74	0.32 - 1.46
5-9	84	3.51	2.80 - 4.34	50	4.02	2.98 - 5.30	34	2.95	2.05 - 4.13
10-14	118	3.72	3.08 - 4.45	76	4.59	3.62 - 5.75	42	2.77	1.99 - 3.74
15-19	320	9.31	8.31 - 10.38	231	12.65	11.07 - 14.39	89	5.52	4.43 - 6.79
20-24	347	11.36	10.19 - 12.62	229	14.09	12.32 - 16.04	119	8.32	6.89 - 9.96
25-29	384	10.85	9.79 - 11.99	210	11.65	10.13 - 13.34	174	10.02	8.59 - 11.63
30-34	479	12.96	11.83 - 14.18	270	14.47	12.79 - 16.30	209	11.43	9.93 - 13.09
35-39	558	13.61	12.51 - 14.79	297	14.42	12.82 - 16.15	261	12.80	11.29 - 14.45
40-44	658	15.93	14.73 - 17.19	348	16.80	15.08 - 18.66	310	15.05	13.42 - 16.82
45-49	747	18.34	17.05 - 19.70	392	19.17	17.32 - 21.17	355	17.50	15.73 - 19.42
50-54	897	23.62	22.10 - 25.21	441	23.36	21.23 - 25.64	456	23.87	21.73 - 26.17
55-59	721	26.06	24.19 - 28.03	339	24.91	22.33 - 27.71	382	27.17	24.51 - 30.04
60-64	651	29.83	27.58 - 32.21	286	27.06	24.01 - 30.38	366	32.53	29.28 - 36.04
65-69	560	30.90	28.40 - 33.57	220	26.40	23.03 - 30.13	339	34.63	31.04 - 38.52
70-74	436	27.84	25.29 - 30.58	174	25.86	22.16 - 30.00	262	29.34	25.89 - 33.11
75-79	266	24.53	21.67 - 27.66	94	22.89	18.49 - 28.01	171	25.38	21.72 - 29.49
80-84	131	22.00	18.39 - 26.10	42	22.58	16.27 - 30.52	89	21.73	17.45 - 26.75
≥85	42	11.46	8.26 - 15.49	14	14.78	8.08 - 24.79	28	10.30	6.84 - 14.88
Total	7 424	15.47	15.12 - 15.83	3 729	15.64	15.14 - 16.15	3 695	15.30	14.81 - 15.80

CI = confidence interval

For peer review only

STROBE Statement—Checklist of items that should be included in reports of ***cohort studies***

	Item No	Recommendation	page
**Title and abstract**	1	(*a*) Indicate the study's design with a commonly used term in the title or the abstract	p1
		(*b*) Provide in the abstract an informative and balanced summary of what was done and what was found	P2
**Introduction**			
Background/rationale	2	Explain the scientific background and rationale for the investigation being reported	P4
Objectives	3	State specific objectives, including any prespecified hypotheses	P4
**Methods**			
Study design	4	Present key elements of study design early in the paper	P5
Setting	5	Describe the setting, locations, and relevant dates, including periods of recruitment, exposure, follow-up, and data collection	P5
Participants	6	(*a*) Give the eligibility criteria, and the sources and methods of selection of participants. Describe methods of follow-up	P5
		(*b*) For matched studies, give matching criteria and number of exposed and unexposed	P5
Variables	7	Clearly define all outcomes, exposures, predictors, potential confounders, and effect modifiers. Give diagnostic criteria, if applicable	P5
Data sources/ measurement	8*	For each variable of interest, give sources of data and details of methods of assessment (measurement). Describe comparability of assessment methods if there is more than one group	P5
Bias	9	Describe any efforts to address potential sources of bias	P6
Study size	10	Explain how the study size was arrived at	P6
Quantitative variables	11	Explain how quantitative variables were handled in the analyses. If applicable, describe which groupings were chosen and why	P6
Statistical methods	12	(*a*) Describe all statistical methods, including those used to control for confounding	P6
		(*b*) Describe any methods used to examine subgroups and interactions	P6
		(*c*) Explain how missing data were addressed	P6
		(*d*) If applicable, explain how loss to follow-up was addressed	
		(*e*) Describe any sensitivity analyses	P6
**Results**			
Participants	13*	(a) Report numbers of individuals at each stage of study—eg numbers potentially eligible, examined for eligibility, confirmed eligible, included in the study, completing follow-up, and analysed	P7
		(b) Give reasons for non-participation at each stage	P7
		(c) Consider use of a flow diagram	NA
Descriptive data	14*	(a) Give characteristics of study participants (eg demographic, clinical, social) and information on exposures and potential confounders	P7
		(b) Indicate number of participants with missing data for each variable of interest	P7
		(c) Summarise follow-up time (eg, average and total amount)	P7
Outcome data	15*	Report numbers of outcome events or summary measures over time	P7
Main results	16	(*a*) Give unadjusted estimates and, if applicable, confounder-adjusted estimates and their precision (eg, 95% confidence interval). Make clear which confounders were adjusted for and why they were included	P7
		(*b*) Report category boundaries when continuous variables were categorized	P7

For peer review only - http://bmjopen.bmj.com/site/about/guidelines.xhtml

		(*c*) If relevant, consider translating estimates of relative risk into absolute risk for a meaningful time period	P7
Other analyses	17	Report other analyses done—eg analyses of subgroups and interactions, and sensitivity analyses	NA
**Discussion**			
Key results	18	Summarise key results with reference to study objectives	P8
Limitations	19	Discuss limitations of the study, taking into account sources of potential bias or imprecision. Discuss both direction and magnitude of any potential bias	P8
Interpretation	20	Give a cautious overall interpretation of results considering objectives, limitations, multiplicity of analyses, results from similar studies, and other relevant evidence	P8
Generalisability	21	Discuss the generalisability (external validity) of the study results	P8
**Other information**			
Funding	22	Give the source of funding and the role of the funders for the present study and, if applicable, for the original study on which the present article is based	P1

*Give information separately for exposed and unexposed groups.

Note: An Explanation and Elaboration article discusses each checklist item and gives methodological background and published examples of transparent reporting. The STROBE checklist is best used in conjunction with this article (freely available on the Web sites of PLoS Medicine at http://www.plosmedicine.org/, Annals of Internal Medicine at http://www.annals.org/, and Epidemiology at http://www.epidem.com/). Information on the STROBE Initiative is available at http://www.strobe-statement.org.

For peer review only

BMJ Open

Four-Year Nationwide Incidence of Retinitis Pigmentosa in South Korea: A Population-based Retrospective Study from 2011 to 2014

Journal:	*BMJ Open*
Manuscript ID	bmjopen-2016-015531.R1
Article Type:	Research
Date Submitted by the Author:	09-Mar-2017
Complete List of Authors:	Rim, Tyler Hyung Taek; Severance Hospital, Institute of Vision Research, Yonsei University College of Medicine, Department of Ophthalmology<br>Park, Hye Won ; Severance Hospital, Institute of Vision Research, Yonsei University College of Medicine, Department of Ophthalmology<br>Kim, Dong Wook ; National Health Insurance Service Ilsan Hospital<br>Chung, Eun Jee ; National Health Insurance Service Ilsan Hospital
**Primary Subject Heading**:	Epidemiology
Secondary Subject Heading:	Ophthalmology, Emergency medicine
Keywords:	retinitis pigmentosa, south korea, Asian population

SCHOLARONE™
Manuscripts

For peer review only

Four-Year Nationwide Incidence of Retinitis Pigmentosa in South Korea: A Population-based Retrospective Study from 2011 to 2014

Tyler Hyungtaek Rim,[1,2*] M.D., M.B.A, Hye Won Park,[1,2*] M.D., Dong Wook Kim,[3] Ph.D., Eun Jee Chung,[1] M.D., Ph.D.

[1]Department of Ophthalmology, National Health Insurance Service Ilsan Hospital, Goyang, Korea; [2]Department of Ophthalmology, Severance Hospital, Institute of Vision Research, Yonsei University College of Medicine, Seoul, Korea; [3]Department of Policy Research Affairs, National Health Insurance Service Ilsan Hospital, Goyang, Gyeonggi-do, Korea;

[*]Contributed equally

Corresponding Author: Eun Jee Chung M.D., Ph.D.

Department of Ophthalmology, National Health Insurance Service Ilsan Hospital

#100 Ilsan-ro, Ilsandong-gu, Goyang 10444, Gyeonggi-do, Korea

Tel: 82-31-900-0590, Fax: 82-31-900-0049

E-mail: eunjee95@nhimc.or.kr

Text Word Count: 2574

Funding Support: This work was supported by a National Health Insurance Ilsan Hospital grant (NHIMC 2015-02-015).

For peer review only

For peer review only - http://bmjopen.bmj.com/site/about/guidelines.xhtml

Abstract

Objective: To determine the incidence of retinitis pigmentosa (RP) in South Korea.

Design: Nationwide, population-based retrospective study.

Setting: Census population of South Korea

Participants: This study involved the entire population of South Korea (n = 47 990 761). Patients confirmed as having RP by an ophthalmologist from 1 January 2011 to 31 December 2014 were included.

Primary outcome measure: The average incidence of RP during the 4-year study period was estimated using population data from the 2010 Korean census.

Results: A total of 3 144 (1 567 men and 1 577 women) patients confirmed as having RP were identified. The average incidence of RP was 1.64 cases/100 000 person-years (95% confidence interval, 1.58–1.70). The incidence of RP distribution skewed to the left across age groups, with one smaller peak observed in the 20–24-year-old age group (1.24 cases/100 000 person-years) and a larger peak observed in the 65–69-year-old age group (3.26 cases/100 000 person-years). The overall incidence was similar in men and women (1.64 cases/100 000 person-years [95% confidence interval, 1.56–1.73] for men; 1.63 cases/100 000 person-years [95% confidence interval, 1.55–1.72] for women).

Conclusions: Our study's estimates of the nationwide population-based incidence of RP in an Asian population will help advance the understanding of the disease onset and allow health care systems to plan accordingly.

For peer review only

For peer review only - http://bmjopen.bmj.com/site/about/guidelines.xhtml

Strengths and limitations of this study

- In this retrospective cohort study of registry data on retinitis pigmentosa (RP) for the entire South Korean population, the incidence of RP was found to be 1.64 cases per 100,000 person-years.
- This study is unique because it included the largest population ever studied in terms of this rare disease, and it represents the first investigation of the nationwide incidence of RP in an Asian country.
- Only a few recent studies have collected data for a general population and calculated the incidence of RP. There are no nationwide RP-related epidemiological studies.
- A lack of clinical information, including family pedigree, visual acuity, visual field, and genetic analysis, is an inherent limitation of the present claims database study.
- Although not ideal, our approach was cost-effective for identifying patients with RP and calculating the incidence of RP in the entire South Korean population.

For peer review only

For peer review only - http://bmjopen.bmj.com/site/about/guidelines.xhtml

Introduction

Retinitis pigmentosa (RP) includes a group of heterogeneous hereditary retinal degeneration disorders caused by the progressive loss of photoreceptor cells.[1 2] It is characterised by widespread retinal degeneration and is an important and leading cause of blindness.[3] However, there is a paucity of data based on large, well-defined populations regarding the epidemiological characteristics of RP. Ideally, a study evaluating the epidemiology of RP should be based on population data rather than on data obtained from a tertiary referral centre to avoid the effects of a referral bias. To the best of our knowledge, only a few recent studies have collected data from a general population and calculated the incidence of RP. The incidence of RP was reported in only two previous studies conducted in Denmark (0.79 cases/100 000 person-years)[4] and Maine in the United State (6 cases/100 000 person-years).[5] Despite recent large-scale health-related studies,[6 7] there are no nationwide RP-related epidemiological studies. In general, incidence estimates provide more information than prevalence estimates with regard to a disease's characteristics and to help health care systems plan accordingly.

South Korea has a mandatory universal health insurance system covering the entire population of 48 million people; therefore, the medical claims database includes all health care use in South Korea. In addition, the Korean government initiated a registration program for rare and intractable disorders, such as RP, in 2007. Patients with RP registered in this program are eligible for up to a 90% co-payment reduction after the diagnosis has been confirmed by an ophthalmologist using the Korean National Health Insurance Service (KNHIS) diagnostic criteria based on an electrophysiological examination. Our study aim was to use this database to conduct a nationwide, population-based study to determine the incidence of RP in South Korea.

For peer review only - http://bmjopen.bmj.com/site/about/guidelines.xhtml

Subjects and Methods

Statement of Ethics

This retrospective nationwide cohort study was reviewed and approved by the Institutional Review Board of the National Health Insurance Service Ilsan Hospital, Gyeonggi-do, Korea. This study adhered to the tenets of the Declaration of Helsinki, and written informed consent was waived.

Retinitis pigmentosa registration in South Korea and its definition

All Korean residents are obligated to enrol in the KNHIS. Claims are accompanied by data regarding diagnostic codes, procedures, prescription drugs, personal information, and information about the hospital. No patient health care records are duplicated because all Korean residents receive a unique identification number at birth. Furthermore, the KNHIS uses the Korean Classification of Diseases, which is a system similar to the International Classification of Diseases. In 2007, the National Health Insurance Service initiated a co-payment reduction of up to 90% for patients suffering from 138 rare and intractable disorders, including RP. Patients with RP who registered in the program were eligible for co-payment reduction after receiving a confirmed diagnosis by an ophthalmologist based on the National Health Insurance Service diagnostic criteria. The National Health Insurance Service diagnostic criteria for RP require (1) ophthalmoscopic abnormalities of the retina on a dilated fundus examination and (2) electroretinographic changes confirming the presence of RP-related photoreceptor damage. All submissions for co-payment reduction registration were reviewed and confirmed by the Health Insurance Review Agency. Patients must reapply for co-payment reduction registration every 5 years after the initial registration to maintain the co-payment reduction. After registration, all RP-related claims contain the RP registration code (V209) in addition to the diagnostic code for RP (Korean Classification of Diseases

For peer review only - http://bmjopen.bmj.com/site/about/guidelines.xhtml

H3551). Patients who filed claims for RP (V209, H3551) from January 2011 to December 2014 with the KNHIS were included in this study. We excluded patients with chronic RP registered between 1 January 2007 and 31 December 2010, because RP (H3551) and Stargardt disease (H3558) shared the same registration code (V209) and did not have a Korean Classification of Diseases diagnostic code in the KNHIS database during this time. Finally, we obtained RP registration data from the national health claim database between 2007 and 2014, and calculated the incidence of RP between 2011 and 2014.

Statistical analysis

The incident time was defined as the registration date of a rare and intractable disorder diagnosed as RP. Annual population data were obtained from the Population and Housing Census conducted in 2010 and available from the Korean Statistical Information Service (http://kosis.kr). Detailed demographic characteristics of the South Korean population are listed in table 1. The person-time incidence rates for 2011–2014 were calculated as the number of people who developed RP divided by the total person-time at risk during the study period. Therefore, in this analysis, person-years were counted after the incident time. The incidence per 100 000 person-years, based on the 2010 census, was estimated using the Poisson distribution. In our explorative analysis (supplementary digital content 1, appendix) the total number of RP cases from 2007 to 2014 was estimated at 7 424. Prevalent cases of RP were not excluded from the population at risk because the denominator (total Korean person-time at risk) was large enough to not be affected in terms of incidence. The total person-time at risk was assumed as the total population (census in 2010, N=47 990 761, table 1) over 4 years from 2011 to 2014 (population multiplied by four). The annual incidence from 2011 to 2014 and overall incidence were estimated with 95% confidence intervals; additionally, the age-specific and sex-specific incidence rates were estimated, and the age-

For peer review only - http://bmjopen.bmj.com/site/about/guidelines.xhtml

specific population for each year was approximated based on the 2010 census population. The male-to-female ratio for the RP incidence rate was also estimated. A significance level of 0.05 was selected. All analyses were conducted using Stata/MP, version 14.0 (StataCorp, College Station, TX, USA).

For peer review only

For peer review only - http://bmjopen.bmj.com/site/about/guidelines.xhtml

Results

The total number of incident cases of RP from 2011 to 2014 in South Korea was 3,144, including 1 567 men (49.9%) and 1 577 women (50.1%). The incidence of RP during the study period was 1.64 cases/100 000 person-years. In men, the incidence was 1.64 cases/100 000 person-years, and in women, it was 1.63 cases/100 000 person-years. The peak incidence in the total population was observed in the 65–69-year-old age group (3.26 cases/100 000 person-years; men, 3.00 cases/100 000 person-years; women, 3.86 cases/100 000 person-years) (table 1 and figure 1). Although there was some variation between the different periods and age groups, the overall incidences in men and women were similar (male-to-female ratio = 1.01, p = 0.80). The incidence of RP distribution was negatively skewed (skewed to the left) across age groups, with one smaller peak observed in 20–24 year olds (1.24 cases/100 000 person-years). The incidence was slightly decreased in the 25–29-year-old age group (1.07 cases/100 000 person-years), and the maximum incidence was seen in the 65–69-year-old age group (3.26 cases/100 000 person-years) (figure 1). The incidence for women steadily increased with age, reaching a maximum level in the 65–69-year-old age group, and then it gradually decreased after age 69. There was a unique peak in incidence for men around 20 years of age (figure 1).

There were 851 (27.1%), 708 (22.5%), 728 (23.2%), and 857 (27.3%) incident cases in 2011, 2012, 2013, and 2014, respectively. There was little difference in the annual number of newly diagnosed patients with RP from 2011 to 2014. The annual incidence of RP was slightly higher in 2011 and 2014, and slightly lower in 2012 and 2013 (1.77 cases/100 000 population in 2011, 1.48 cases/100 000 population in 2012, 1.52 cases/100 000 population in 2013, and 1.79 cases/100 000 population in 2014, table 2).

For peer review only

For peer review only - http://bmjopen.bmj.com/site/about/guidelines.xhtml

Discussion

In the present study, using the KNHIS database between 2011 and 2014, we found a nationwide, population-based, estimated RP incidence of 1.64 cases/100 000 person-years. This study involved a survey of the entire South Korean population using reliable data based on an ophthalmologist-confirmed RP registration.

RP is not an acute symptomatic disease like retinal vascular occlusion. The most common initial symptom of RP, night blindness, has an insidious onset and may go unperceived for many years. Patients often become cognisant of RP incidentally. Therefore, previous studies from Japan noted the disease onset as the time of a diagnosis of RP by an ophthalmologist.[8] Another study from Denmark discussed possible recall bias, and patients were classified as possible, probable, or certain according to the presence of specified diagnostic criteria and confirmatory electroretinographic changes. RP is very rare; therefore, it is impossible to estimate the incidence without a large cohort of subjects, such as that provided by nationwide data. However, it is practically impossible to perform ophthalmological examinations of the entire population to diagnosis a rare intractable disease such as RP. Our study design was based on the ophthalmologist-confirmed RP registry, and it demonstrated a high specificity for the diagnosis. Although not ideal, our approach was cost-effective for identifying patients with RP and calculating the incidence of RP in the entire South Korean population. Studying a large cohort, such as the entire South Korean population, and having a 4-year study period may provide stability to the heterogeneous detection rate seen with this insidious disease. These properties limit the chance probabilities that may occur with studies of smaller localised populations or tertiary hospital-based populations. Studies regarding the incidence of other rare intractable diseases, as exudative age-related macular degeneration[6] and Moyamoya disease,[7] using the same registration database, have been previously reported.

For peer review only

Using the South Korean national claims database from 2011 to 2014, which includes the RP registration program, we estimated the incidence rate of RP as 1.64 cases/100 000 person-years (95% confidence interval, 1.58–1.70). To the best of our knowledge, only two previous studies have estimated the incidence of RP based on an adequate number of patients with RP. A well-defined local area study conducted in the 1980s in the state of Maine (USA) estimated that the incidence of RP was 6 cases/100 000 person-years.[5] Another important previous study regarding the epidemiology of RP, performed in Denmark in the 2000s, reported an average incidence of 0.79 cases/100 000 person-years from 1990 to 1997.[4] The incidence of RP in South Korea found in the present study is lower than that seen in Maine and greater than that seen in Denmark.

Information regarding the age of disease onset is meaningful in genetic counselling, and a previous study from Japan described an age at onset curve for RP based on 370 patients with RP.[8] The age at onset, defined as the age of diagnosis, gradually increased with age until age 65 and subsequently remained steady after the age of 65.[8] In the Danish RP study,[4] the incidence of RP also increased until age 60–64 years, and then it decreased in people older than 65 years old, similar to the trend seen in the present study (figure 1). In terms of sex, the Danish cohorts with RP showed a nearly equal increase in the annual incidence between men and women from 1990 to 1997.[4] The present study showed similar trends; the overall male-to-female ratio was 1.01 ($p = 0.86$), and the annual incidence of RP was similar for men and women during the 4-year study period (table 2). However, the estimated age-specific incidence of newly diagnosed patients was nearly double in male patients compared to female patients in the 20–24-year-old age group (men, 1.54 cases/100 000 population and women, 0.89 cases/100 000 population) (table 1). In addition, the incidence of RP was greater in women than in men aged 50–65 years. In the Danish RP study, the age at onset was much

For peer review only - http://bmjopen.bmj.com/site/about/guidelines.xhtml

higher in male patients than in female patients aged 6–18 years.[4] Our results also show that the onset may be earlier in male individuals than in female individuals. However, the specifics of military service in South Korea should also be considered. The higher incidence of RP in men in their 20s entering mandatory military service in South Korea may be because they are more frequently subjected to physical examinations related to potential exemption from the military service.

To the best of our knowledge, this is the largest population ever studied for this rare disease based on an ophthalmologist-confirmed diagnosis. It is also the first study to investigate the nationwide incidence of RP in an Asian country.

However, there are several limitations of this study. First, as aforementioned, it is difficult to define the exact time of onset of RP, and we arbitrary defined the time of occurrence of RP as the time when the patients were diagnosed as having RP by an ophthalmologist, as in previous studies.[4 8] Second, we may have underestimated the incidence rates of RP. We identified patients with RP using health care claims but could not include asymptomatic patients or patients who did not use health care services. Since there is no cure for RP yet, some patients may not want to register, even though they have been diagnosed as having RP. However, it is reasonable to assume that people with symptomatic RP are likely to use health care services at some time in the course of the disease. The universal health insurance coverage and co-payment reduction with the registration program for this rare and intractable disorder may also help to encourage health care use among patients with RP. Moreover, health care accessibility in South Korea is very high. The estimated incidence of newly diagnosed patients showed a consistent annual pattern, and the relatively long study period, including 4 years of data, may offset this limitation. Third, the diagnosis of RP was defined based on the health care registration and a Korean Classification of Diseases code, which

For peer review only - http://bmjopen.bmj.com/site/about/guidelines.xhtml

may be inaccurate compared with a diagnosis obtained from a medical record. However, patients with RP in this study had their RP diagnoses confirmed by ophthalmologists based on the National Health Insurance Service diagnostic criteria, including electroretinography, which provides a test result that is considered a confirmatory criterion.[4] In addition, the eligibility of submitted registrations was reviewed comprehensively by the Health Insurance Review Agency. Fourth, a lack of clinical information, including family pedigree, visual acuity, visual field, and genetic analysis, is another inherent limitation of the present claims database study. Fifth, we only targeted subjects in South Korea. Therefore, this result cannot be directly compared with those of other ethnic groups. Lastly, we did not report the prevalence of RP because of the characteristics of the South Korean national claims database. The analysis of prevalence for RP was impossible with the Korean Classification of Disease, sixth edition (Korean Classification of Diseases-6) system, in which RP, Stargardt disease, vitelliform retinal dystrophy, and other unspecified hereditary retinal dystrophies were similarly categorised as hereditary retinal dystrophy (H35.5). In this study, we excluded subjects with RP registered prior to December 31, 2010, because it was difficult to distinguish among these diseases in the KNHIS database. In our explorative analysis, only 109 incident cases of Stargardt disease were observed from 2011 to 2014, a relatively small number compared to the 3 144 cases of RP. Therefore, we have presented the period prevalence of presumed RP in the supplementary digital content 1 of the appendix as 15.47 cases/100 000 persons (~1/6500), a value consistent with previous reports (12–26 cases/100 000 persons or 1/3800 – 8300)[5 9-11] and slightly lower than that found in Danish cohorts with RP (22.4 cases/100 000 persons).[4]

In conclusion, we estimated the nationwide incidence of newly diagnosed patients with RP in South Korea using a database that included the entire national population during the 4-year

For peer review only - http://bmjopen.bmj.com/site/about/guidelines.xhtml

period from 2011 to 2014. The estimated population-based incidence rate of RP for all ages was 1.64 cases/100 000 persons-years, and there was generally no difference in the rates between men and women. Our study should be helpful in assessing the RP-related socioeconomic burden and in planning accordingly within the health care system.

For peer review only

For peer review only - http://bmjopen.bmj.com/site/about/guidelines.xhtml

Acknowledgments

This work was supported by a National Health Insurance Ilsan Hospital grant (NHIMC 2015-02-015). This study used data from the NHIS-NCS 2002-2013 (NHIS-2015-1-068), which was released by the KNHIS. The authors alone are responsible for the content and writing of this article. Eun Jee Chung had full access to all of the data in the study, and takes responsibility for the integrity of the data and the accuracy of the data analysis.

Competing Interests: We have read and understood BMJ policy on declaration of interests and declare that we have no competing interests.

Data sharing statement: Access to NHIS-NSC data are available from the website of NHIS (https://nhiss.nhis.or.kr) after completing the application process and receiving approval (http://nhiss.nhis.or.kr/bd/ab/bdaba021eng.do). Detailed cohort profile and the methods for obtaining data are explained in the following source: Lee J, Lee JS, Park SH, Shin SA, Kim K. Cohort Profile: The National Health Insurance Service-National Sample Cohort (NHIS-NSC), South Korea. International journal of epidemiology. 2016. doi: 10.1093/ije/dyv319. PubMed PMID: 26822938.

Authors' Contributions:

Conception and design: THR and EJC

Analysis and interpretation: THR, HWP, DWK, and EJC

Data collection: THR, HWP, DWK, and EJC

Manuscript preparation: THR and HWP

Overall responsibility: EJC

For peer review only - http://bmjopen.bmj.com/site/about/guidelines.xhtml

References

1. Carr RE, Noble KG. Retinitis pigmentosa. *Ophthalmology* 1981;88(2):169-72.
2. Hartong DT, Berson EL, Dryja TP. Retinitis pigmentosa. *Lancet* 2006;368(9549):1795-809. doi: 10.1016/S0140-6736(06)69740-7
3. Buch H, Vinding T, La Cour M, et al. Prevalence and causes of visual impairment and blindness among 9980 Scandinavian adults: the Copenhagen City Eye Study. *Ophthalmology* 2004;111(1):53-61.
4. Haim M. Epidemiology of retinitis pigmentosa in Denmark. *Acta Ophthalmol Scand Suppl* 2002(233):1-34.
5. Bunker CH, Berson EL, Bromley WC, et al. Prevalence of retinitis pigmentosa in Maine. *Am J Ophthalmol* 1984;97(3):357-65.
6. Park SJ, Kwon KE, Choi NK, et al. Prevalence and Incidence of Exudative Age-Related Macular Degeneration in South Korea: A Nationwide Population-Based Study. *Ophthalmology* 2015;122(10):2063-70 e1. doi: 10.1016/j.ophtha.2015.06.018
7. Ahn IM, Park DH, Hann HJ, et al. Incidence, prevalence, and survival of moyamoya disease in Korea: a nationwide, population-based study. *Stroke* 2014;45(4):1090-5. doi: 10.1161/STROKEAHA.113.004273
8. Tsujikawa M, Wada Y, Sukegawa M, et al. Age at onset curves of retinitis pigmentosa. *Archives of ophthalmology* 2008;126(3):337-40.
9. Bundey S, Crews SJ. A study of retinitis pigmentosa in the City of Birmingham. I Prevalence. *J Med Genet* 1984;21(6):417-20.
10. Grondahl J. Tapeto-retinal degeneration in four Norwegian counties, II. Diagnostic evaluation of 407 relatives and genetic evaluation of 87 families. *Clin Genet* 1986;29(1):17-41.
11. Hu DN. Prevalence and mode of inheritance of major genetic eye diseases in China. *J Med Genet* 1987;24(10):584-8.

For peer review only

For peer review only - http://bmjopen.bmj.com/site/about/guidelines.xhtml

Figure Legend

Figure 1. Average incidence of retinitis pigmentosa (RP). Incidence per 100 000 person-years of RP in the South Korean population according to age groups from 2011 to 2014.

Supplementary Information

Supplementary Digital Content 1, Appendix (Text Document)

For peer review only

For peer review only - http://bmjopen.bmj.com/site/about/guidelines.xhtml

Table 1 Demographic characteristics of South Korea and the incidence of retinitis pigmentosa per 100 000 person-years in the South Korean population (2011-2014)

	Number of Koreans			Total			Men			Women			Male-to-	P value
Age (years)	Total	Men	Women	No.	Incidence (95% CI)		No.	Incidence (95% CI)		No.	Incidence (95% CI)		female Ratio	(chi test)
0-4	2 219 084	1 142 220	1 076 864	4	0.05	0.01- 0.12	4	0.09	0.02- 0.22	0	no observation		NA	0.05
5-9	2 394 663	1 243 294	1 151 369	21	0.22	0.14- 0.34	14	0.28	0.15- 0.47	7	0.15	0.06- 0.31	1.86	0.18
10-14	3 173 226	1 654 964	1 518 262	28	0.22	0.15- 0.32	16	0.24	0.14- 0.39	12	0.20	0.10- 0.35	1.22	0.60
15-19	3 438 414	1 826 179	1 612 235	123	0.89	0.74- 1.07	92	1.26	1.02- 1.54	31	0.48	0.33- 0.68	2.62	<0.05
20-24	3 055 420	1 625 371	1 430 049	151	1.24	1.05- 1.45	100	1.54	1.25- 1.87	51	0.89	0.66- 1.17	1.73	<0.05
25-29	3 538 949	1 802 805	1 736 144	152	1.07	0.91- 1.26	84	1.16	0.93- 1.44	68	0.98	0.76- 1.24	1.18	0.29
30-34	3 695 348	1 866 397	1 828 951	209	1.41	1.23- 1.62	112	1.50	1.24- 1.81	97	1.33	1.08- 1.62	1.13	0.37
35-39	4 099 147	2 060 233	2 038 914	239	1.46	1.28- 1.65	129	1.57	1.31- 1.86	110	1.34	1.10- 1.61	1.17	0.25
40-44	4 131 423	2 071 431	2 059 992	307	1.86	1.66- 2.08	162	1.96	1.67- 2.28	145	1.76	1.48- 2.07	1.11	0.36
45-49	4 073 358	2 044 641	2 028 717	304	1.87	1.66- 2.09	148	1.81	1.53- 2.13	156	1.92	1.63- 2.25	0.94	0.60
50-54	3 798 131	1 887 973	1 910 158	405	2.67	2.41- 2.94	204	2.70	2.34- 3.10	201	2.63	2.28- 3.02	1.03	0.79
55-59	2 766 695	1 360 747	1 405 948	327	2.95	2.64- 3.29	150	2.76	2.33- 3.23	177	3.15	2.70- 3.65	0.88	0.23
60-64	2 182 236	1 057 035	1 125 201	283	3.24	2.88- 3.64	127	3.00	2.50- 3.57	156	3.47	2.94- 4.05	0.86	0.23
65-69	1 812 168	833 242	978 926	236	3.26	2.85- 3.70	85	2.55	2.04- 3.15	151	3.86	3.27- 4.52	0.66	<0.05
70-74	1 566 014	672 894	893 120	178	2.84	2.44- 3.29	72	2.68	2.09- 3.37	106	2.97	2.43- 3.59	0.90	0.50
75-79	1 084 367	410 726	673 641	108	2.49	2.04- 3.01	49	2.98	2.21- 3.94	59	2.19	1.67- 2.82	1.36	0.11
80-84	595 509	186 008	409 501	56	2.35	1.78- 3.05	13	1.75	0.93- 2.99	43	2.63	1.90- 3.54	0.67	0.20
≥85	366 609	94 736	271 873	13	0.89	0.47- 1.52	6	1.58	0.58- 3.45	7	0.64	0.26- 1.33	2.45	0.09
Total	47 990 761	23 840 896	24 149 865	3144	1.64	1.58- 1.70	1567	1.64	1.56- 1.73	1577	1.63	1.55- 1.72	1.01	0.86

The population of Korea was based on the 2010 census from the Korean Statistical Information Service. NA = not available, CI = confidence interval.

For peer review only - http://bmjopen.bmj.com/site/about/guidelines.xhtml

Table 2 Demographic characteristics of South Koreans and the annual incidence of retinitis pigmentosa per 100 000 person-years in the South Korean population (2011-2014)

	2011			2012			2013			2014		
Age (years)	Total	Men	Women	Total	Men	Women	Total	Men	Women	Total	Men	Women
0-4	0.05	0.09	0.00	0.05	0.09	0.00	0.05	0.09	0.00	0.05	0.09	0.00
5-9	0.21	0.40	0.00	0.33	0.40	0.26	0.17	0.08	0.26	0.17	0.24	0.09
10-14	0.13	0.24	0.00	0.25	0.18	0.33	0.25	0.36	0.13	0.25	0.18	0.33
15-19	1.16	1.75	0.50	0.84	1.15	0.50	0.73	0.88	0.56	0.84	1.26	0.37
20-24	1.44	1.66	1.19	0.88	1.11	0.63	1.15	1.48	0.77	1.47	1.91	0.98
25-29	1.47	1.61	1.32	0.88	0.94	0.81	0.93	1.22	0.63	1.02	0.89	1.15
30-34	1.41	1.23	1.59	1.19	1.34	1.04	1.49	1.71	1.26	1.57	1.71	1.42
35-39	1.68	1.84	1.52	1.22	1.26	1.18	1.59	1.70	1.47	1.34	1.46	1.23
40-44	1.65	1.54	1.75	1.94	2.17	1.70	1.69	1.79	1.60	2.15	2.32	1.99
45-49	1.77	1.71	1.82	1.69	1.91	1.48	1.72	1.37	2.07	2.28	2.25	2.32
50-54	3.37	3.07	3.66	2.47	2.65	2.30	2.24	2.28	2.20	2.58	2.81	2.36
55-59	3.04	3.01	3.06	2.42	2.35	2.49	2.82	2.79	2.85	3.54	2.87	4.20
60-64	3.39	2.93	3.82	3.30	3.41	3.20	3.07	2.74	3.38	3.21	2.93	3.47
65-69	3.92	2.76	4.90	2.70	2.28	3.06	2.98	1.92	3.88	3.42	3.24	3.58
70-74	3.00	2.97	3.02	2.68	3.27	2.24	2.81	2.08	3.36	2.87	2.38	3.25
75-79	2.21	2.92	1.78	1.75	2.19	1.48	1.94	2.43	1.63	4.06	4.38	3.86
80-84	2.02	1.08	2.44	2.35	0.54	3.17	2.02	3.23	1.47	3.02	2.15	3.42
≥85	1.09	2.11	0.74	1.09	2.11	0.74	0.27	0.00	0.37	1.09	2.11	0.74
Total	1.77	1.74	1.81	1.48	1.56	1.40	1.52	1.50	1.53	1.79	1.77	1.80

For peer review only - http://bmjopen.bmj.com/site/about/guidelines.xhtml

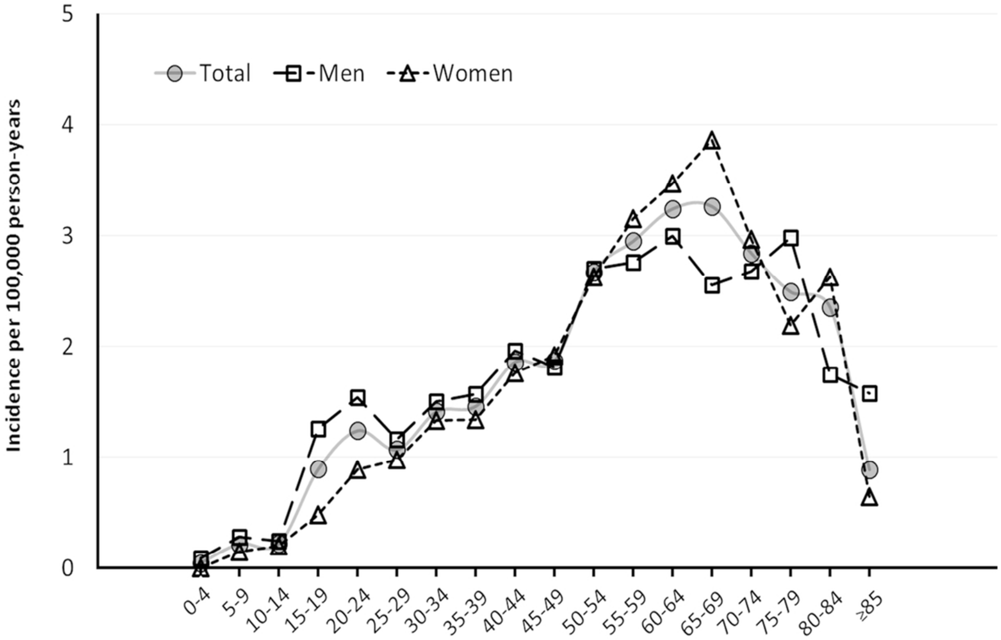

Figure 1. Average incidence of retinitis pigmentosa (RP). Incidence per 100 000 person-years of RP in the South Korean population according to age groups from 2011 to 2014.

51x33mm (600 x 600 DPI)

Prevalence of Presumed Retinitis Pigmentosa in South Korea

The Korean Classification of Disease, version 5 was used until 2010, and its classification system rendered it impossible to distinguish Stargardt disease, a disease that shares the same co-payment policy code (V209), from retinitis pigmentosa (RP). Beginning in 2011, the Korean Classification of Disease, version 6 was used, and it enables the discrimination of these two diseases based on the diagnostic code used (H35.51 for RP and H35.58 for Stargardt disease). Therefore, we excluded patients with chronic RP or Stargardt disease from the 2007 (the first year that the co-payment policy started) through 2010 data, and new cases from 1 January 2011 were evaluated to determine the incidence of RP. In our exploratory analysis, there were 27 patients with Stargardt disease diagnosed in 2011, 26 diagnosed in 2012, 29 diagnosed in 2012, and 27 diagnosed in 2014 in South Korea. One hundred nine patients with Stargardt disease was relatively small compared to the 3144 patients with RP diagnosed over the same period. Therefore, to calculate the prevalence, we arbitrarily defined the presumed number of patients with RP between 2007 and 2010 based on the aforementioned incidence ratio of 109/3144. The period prevalence of presumed RP per 100 000 persons was estimated based on data from the Population and Housing Census conducted in 2010.

Overall, 4 428 patients with RP and Stargardt disease were identified between 2007 and 2010, and the presumed number of patients with RP was calculated to be 4 280 [= 4 428 × 3 144 / (3 144 + 109)]. Among 4 428 patients identified as having RP and Stargardt disease, 863 presumed patients with RP were diagnosed in 2007, 1359 in 2008, 1 228 in 2009, and 978 in 2010. As the co-payment policy was established in 2007 and chronic patients as well as new patients started to register from 2007, there was a larger number of patients registered in 2008 and 2009 than in 2010. A total of 3 144 patients with RP were identified between 2011 and 2014. Finally, 7 424 presumed patients with RP (n = 4 280) and RP (n = 3 144) were identified and used to estimate the prevalence. The prevalence of presumed RP was 15.47 cases per 100 000 persons from 2007 to 2014 (table S1). The prevalence was similar between men (15.64 cases/100 000 persons) and women (15.30 cases/100 000 persons). The 50–54-year-old age group had the largest number of patients with RP with 897 prevalent cases. However, the age-specific prevalence was highest in the 65–69-year-old age group with 30.90 cases/100 000 persons.

In the 1990s, several investigators reported an RP prevalence ranging from 12 (1:8247) to 26 cases (1:3784) per 100 000 persons based on well-defined population studies.[1-4] The RP prevalence in the present study, 15.47 cases/100 000 persons, was smaller than that seen in the Danish cohort with RP (22.4 cases/100 000 persons). However, trends in the age-specific prevalence were similar, showing increasing prevalence with age until 70 years old and then a decreasing prevalence.[5] Other recent studies have also reported prevalence; however, some were limited because of the small number of patients with RP.[6-9] In summary, the prevalence of RP, including presumed patients with RP diagnosed between 2007 and 2010, and RP diagnosed between 2011 and 2014 was 15.47 cases/100 000 (1/6500) persons. These data are within the prevalence range reported in previous well-designed studies and slightly lower than that observed in the Danish cohort with RP.

References

1. Bunker CH, Berson EL, Bromley WC, Hayes RP, Roderick TH. Prevalence of retinitis pigmentosa in Maine. Am J Ophthalmol. 1984;97(3):357-365.
2. Bundey S, Crews SJ. A study of retinitis pigmentosa in the City of Birmingham. I Prevalence. J Med Genet. 1984;21(6):417-420.
3. Grondahl J. Tapeto-retinal degeneration in four Norwegian counties, II. Diagnostic evaluation of 407 relatives and genetic evaluation of 87 families. Clin Genet. 1986;29(1):17-41.
4. Hu DN. Prevalence and mode of inheritance of major genetic eye diseases in China. J Med Genet. 1987;24(10):584-588.
5. Haim M. Epidemiology of retinitis pigmentosa in Denmark. Acta Ophthalmol Scand Suppl. 2002(233):1-34.
6. You QS, Xu L, Wang YX, et al. Prevalence of retinitis pigmentosa in North China: the Beijing Eye Public Health Care Project. Acta Ophthalmol. 2013;91(6):e499-500.
7. Nangia V, Jonas JB, Khare A, Sinha A. Prevalence of retinitis pigmentosa in India: the Central India Eye and Medical Study. Acta Ophthalmol. 2012;90(8):e649-650.
8. Xu L, Hu L, Ma K, Li J, Jonas JB. Prevalence of retinitis pigmentosa in urban and rural adult Chinese: The Beijing Eye Study. Eur J Ophthalmol. 2006;16(6):865-866.
9. Sen P, Bhargava A, George R, et al. Prevalence of retinitis pigmentosa in South Indian population aged above 40 years. Ophthalmic Epidemiol. 2008;15(4):279-281.

Table S1 Period Prevalence Per 100 000 Persons of Presumed Retinitis Pigmentosa in the Korean Population from 2007 to 2014

	Total			Men			Women		
Age (years)	No.	Prevalence (95% CI)		No.	Prevalence (95% CI)		No.	Prevalence (95% CI)	
0-4	25	1.13	0.73 - 1.66	18	1.58	0.93 - 2.49	8	0.74	0.32 - 1.46
5-9	84	3.51	2.80 - 4.34	50	4.02	2.98 - 5.30	34	2.95	2.05 - 4.13
10-14	118	3.72	3.08 - 4.45	76	4.59	3.62 - 5.75	42	2.77	1.99 - 3.74
15-19	320	9.31	8.31 - 10.38	231	12.65	11.07 - 14.39	89	5.52	4.43 - 6.79
20-24	347	11.36	10.19 - 12.62	229	14.09	12.32 - 16.04	119	8.32	6.89 - 9.96
25-29	384	10.85	9.79 - 11.99	210	11.65	10.13 - 13.34	174	10.02	8.59 - 11.63
30-34	479	12.96	11.83 - 14.18	270	14.47	12.79 - 16.30	209	11.43	9.93 - 13.09
35-39	558	13.61	12.51 - 14.79	297	14.42	12.82 - 16.15	261	12.80	11.29 - 14.45
40-44	658	15.93	14.73 - 17.19	348	16.80	15.08 - 18.66	310	15.05	13.42 - 16.82
45-49	747	18.34	17.05 - 19.70	392	19.17	17.32 - 21.17	355	17.50	15.73 - 19.42
50-54	897	23.62	22.10 - 25.21	441	23.36	21.23 - 25.64	456	23.87	21.73 - 26.17
55-59	721	26.06	24.19 - 28.03	339	24.91	22.33 - 27.71	382	27.17	24.51 - 30.04
60-64	651	29.83	27.58 - 32.21	286	27.06	24.01 - 30.38	366	32.53	29.28 - 36.04
65-69	560	30.90	28.40 - 33.57	220	26.40	23.03 - 30.13	339	34.63	31.04 - 38.52
70-74	436	27.84	25.29 - 30.58	174	25.86	22.16 - 30.00	262	29.34	25.89 - 33.11
75-79	266	24.53	21.67 - 27.66	94	22.89	18.49 - 28.01	171	25.38	21.72 - 29.49
80-84	131	22.00	18.39 - 26.10	42	22.58	16.27 - 30.52	89	21.73	17.45 - 26.75
≥85	42	11.46	8.26 - 15.49	14	14.78	8.08 - 24.79	28	10.30	6.84 - 14.88
Total	7 424	15.47	15.12 - 15.83	3 729	15.64	15.14 - 16.15	3 695	15.30	14.81 - 15.80

CI = confidence interval; no. = number

For peer review only

STROBE Statement—Checklist of items that should be included in reports of ***cohort studies***

	Item No	Recommendation	page
**Title and abstract**	1	(*a*) Indicate the study's design with a commonly used term in the title or the abstract	p1
		(*b*) Provide in the abstract an informative and balanced summary of what was done and what was found	P2
**Introduction**			
Background/rationale	2	Explain the scientific background and rationale for the investigation being reported	P4
Objectives	3	State specific objectives, including any prespecified hypotheses	P4
**Methods**			
Study design	4	Present key elements of study design early in the paper	P5
Setting	5	Describe the setting, locations, and relevant dates, including periods of recruitment, exposure, follow-up, and data collection	P5
Participants	6	(*a*) Give the eligibility criteria, and the sources and methods of selection of participants. Describe methods of follow-up	P5
		(*b*) For matched studies, give matching criteria and number of exposed and unexposed	P5
Variables	7	Clearly define all outcomes, exposures, predictors, potential confounders, and effect modifiers. Give diagnostic criteria, if applicable	P5
Data sources/ measurement	8*	For each variable of interest, give sources of data and details of methods of assessment (measurement). Describe comparability of assessment methods if there is more than one group	P5
Bias	9	Describe any efforts to address potential sources of bias	P6
Study size	10	Explain how the study size was arrived at	P6
Quantitative variables	11	Explain how quantitative variables were handled in the analyses. If applicable, describe which groupings were chosen and why	P6
Statistical methods	12	(*a*) Describe all statistical methods, including those used to control for confounding	P6
		(*b*) Describe any methods used to examine subgroups and interactions	P6
		(*c*) Explain how missing data were addressed	P6
		(*d*) If applicable, explain how loss to follow-up was addressed	
		(*e*) Describe any sensitivity analyses	P6
**Results**			
Participants	13*	(a) Report numbers of individuals at each stage of study—eg numbers potentially eligible, examined for eligibility, confirmed eligible, included in the study, completing follow-up, and analysed	P7
		(b) Give reasons for non-participation at each stage	P7
		(c) Consider use of a flow diagram	NA
Descriptive data	14*	(a) Give characteristics of study participants (eg demographic, clinical, social) and information on exposures and potential confounders	P7
		(b) Indicate number of participants with missing data for each variable of interest	P7
		(c) Summarise follow-up time (eg, average and total amount)	P7
Outcome data	15*	Report numbers of outcome events or summary measures over time	P7
Main results	16	(*a*) Give unadjusted estimates and, if applicable, confounder-adjusted estimates and their precision (eg, 95% confidence interval). Make clear which confounders were adjusted for and why they were included	P7
		(*b*) Report category boundaries when continuous variables were categorized	P7

For peer review only - http://bmjopen.bmj.com/site/about/guidelines.xhtml

		(*c*) If relevant, consider translating estimates of relative risk into absolute risk for a meaningful time period	P7
Other analyses	17	Report other analyses done—eg analyses of subgroups and interactions, and sensitivity analyses	NA
**Discussion**			
Key results	18	Summarise key results with reference to study objectives	P8
Limitations	19	Discuss limitations of the study, taking into account sources of potential bias or imprecision. Discuss both direction and magnitude of any potential bias	P8
Interpretation	20	Give a cautious overall interpretation of results considering objectives, limitations, multiplicity of analyses, results from similar studies, and other relevant evidence	P8
Generalisability	21	Discuss the generalisability (external validity) of the study results	P8
**Other information**			
Funding	22	Give the source of funding and the role of the funders for the present study and, if applicable, for the original study on which the present article is based	P1

*Give information separately for exposed and unexposed groups.

Note: An Explanation and Elaboration article discusses each checklist item and gives methodological background and published examples of transparent reporting. The STROBE checklist is best used in conjunction with this article (freely available on the Web sites of PLoS Medicine at http://www.plosmedicine.org/, Annals of Internal Medicine at http://www.annals.org/, and Epidemiology at http://www.epidem.com/). Information on the STROBE Initiative is available at http://www.strobe-statement.org.

For peer review only
